# Culprit or Innocent Bystander? A Case of Hematemesis

**DOI:** 10.1097/PG9.0000000000000263

**Published:** 2022-10-20

**Authors:** Prathipa Santhanam, Alina Iuga, Francisco A. Sylvester

**Affiliations:** From the * Department of Pediatric Gastroenterology, North Carolina Children’s Hospital, Chapel Hill, NC; † Department of Pathology and Laboratory Medicine, Chapel Hill, NC.

An 11-year-old previously healthy female presented with 1 year history of generalized abdominal pain, early satiety and intermittent hematemesis without history of melena. She underwent esophagogastroduodenoscopy (EGD) as a part of her workup. EGD was notable for diffuse gastric erythema and retained food in stomach despite being nil per os for solid food for 14 hours (Fig. 1). Histopathologic examination showed chronic gastritis in the gastric body and focal organisms identified morphologically as *Sarcina ventriculi* (Fig. 2).

**FIGURE 1.**
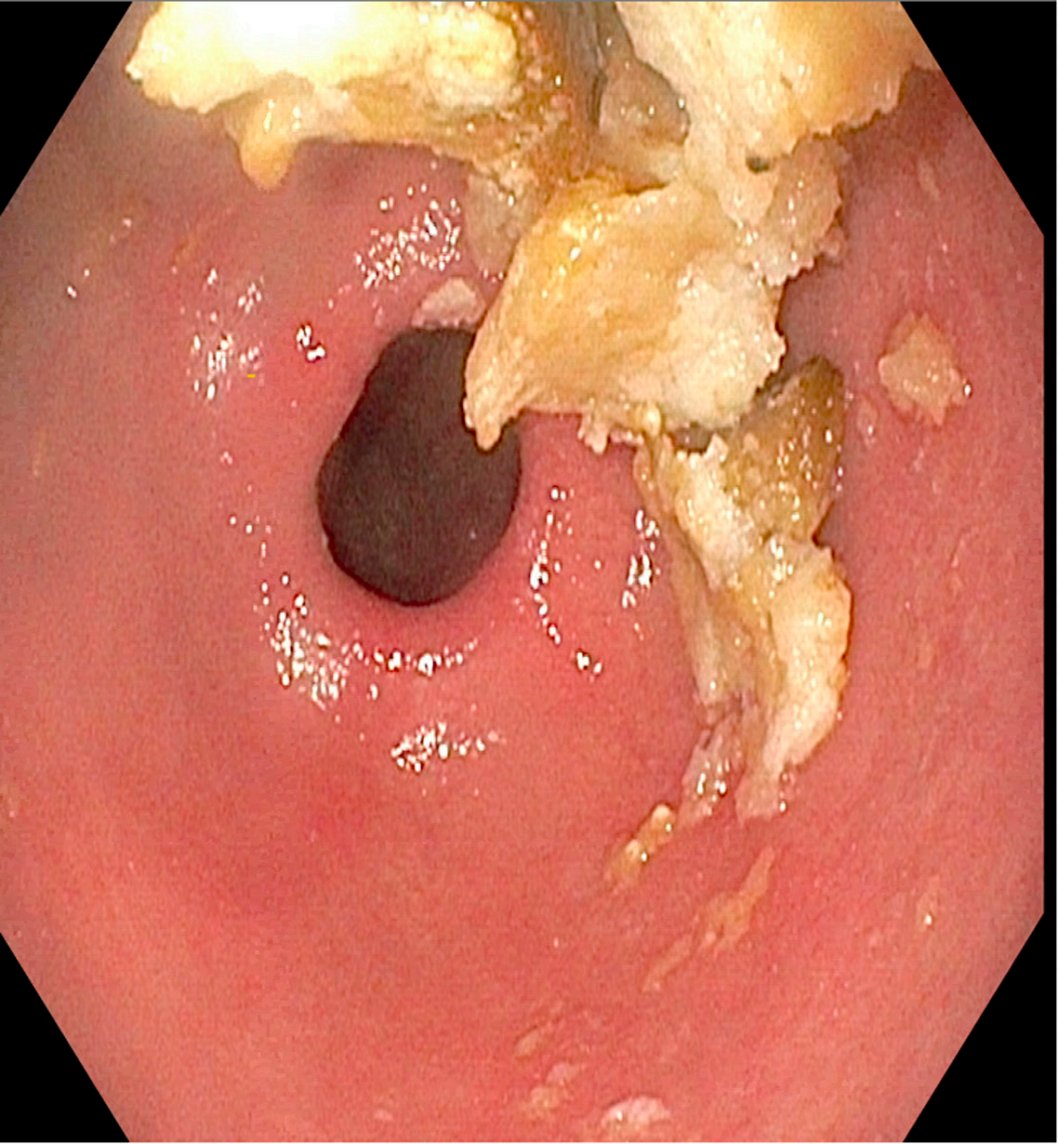
Gastric mucosal erythema and retained food in stomach.

**FIGURE 2.**
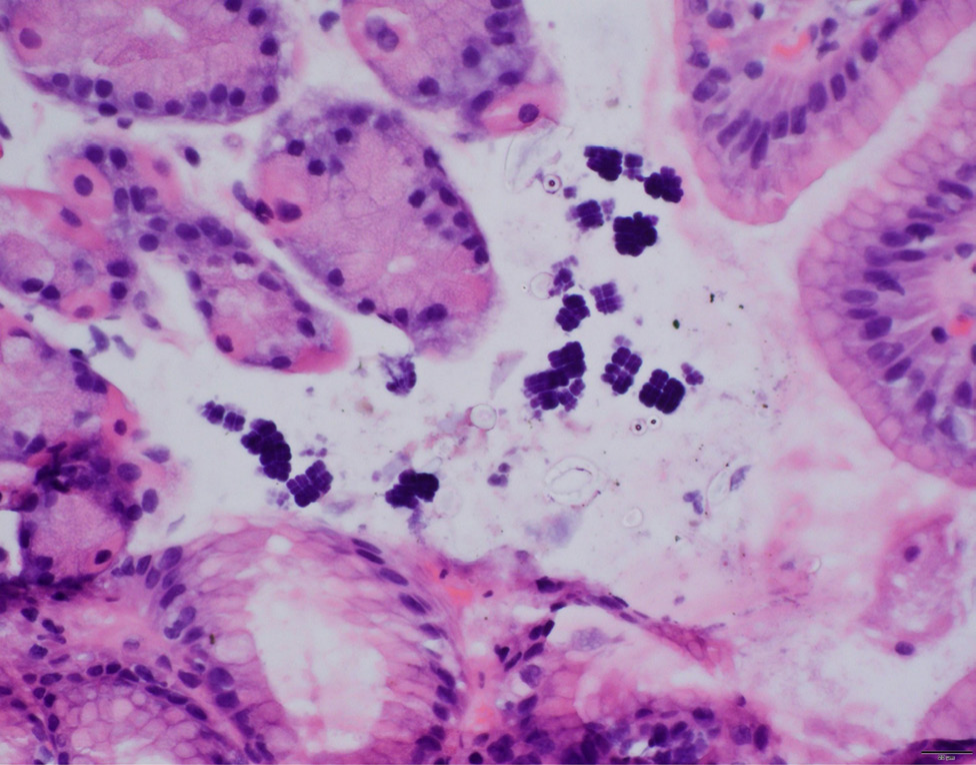
Photomicrograph showing *S. ventriculi* organisms in the stomach mucosa with characteristic morphology: Aggregates of basophilic stained, cuboid organisms in tetrad packet arrangement with flattening of the cell walls in areas of contact with adjacent cells. Hematoxylin and eosin stain, original magnification ×40.

*S. ventriculi* is a Gram-positive anaerobic coccus with a birefringent cellulose coating, averaging from 1.8 to 3 μm in size, that is found in water and soil and thrives in low pH environment. In the stomach, it appears as clusters of tetrads. Its metabolism is strictly carbohydrate dependent. It is unclear if *S ventriculi* has a pathogenic role, but it has been associated with gastroparesis, gastric ulcers, mucosal inflammation (1), emphysematous gastritis (2), gastric perforation (3), and esophagitis (4). Local accumulation of acetaldehyde and ethanol from carbohydrate fermentation by *S. ventriculi* may cause gastric mucosal injury. The most common endoscopic feature (5), which was found in this patient, is the presence of food retention due to delayed gastric emptying, often accompanied by an inflamed gastric mucosa.

There are neither consensus on which patients need treatment nor guidelines regarding antibiotic selection or duration of treatment. In this case, the child received empiric treatment with metronidazole and amoxicillin/clavulanic acid for 1 week along with a proton-pump inhibitor for 2 weeks, with resolution of hematemesis and improvement of her abdominal pain.

## ACKNOWLEDGMENTS

Informed consent was provided by patient’s parents for this report to be published
